# Short‐term mortality among very elderly cancer patients in the intensive care unit: A retrospective cohort study based on the Medical Information Mart for Intensive Care IV database

**DOI:** 10.1002/agm2.12358

**Published:** 2024-10-18

**Authors:** Taotao Liu, Runyu Ding

**Affiliations:** ^1^ Department of Surgical Intensive Care Unit, Beijing Hospital, National Center of Gerontology, Institute of Geriatric Medicine Chinese Academy of Medical Sciences Beijing China; ^2^ Department of Surgical Intensive Care Unit, Fuwai Hospital, National Center of Cardiovascular Diseases Chinese Academy of Medical Sciences and Peking Union Medical College Beijing China

**Keywords:** aged, 80 and over, database, factual, malignant neoplasm, mortality, severity of illness index

## Abstract

**Objective:**

The objective of this study is to examine the epidemiological characteristics of very elderly patients (aged over 80 years) with cancer admitted to the intensive care unit (ICU), and to elucidate the association between Acute Physiology Score III (APS‐III) and 28‐day mortality.

**Method:**

A retrospective analysis was conducted using data extracted from the Medical Information Mart for Intensive Care IV (MIMIC‐IV) database. Patients aged 80 years and above were assigned to three groups: non‐cancer group, non‐metastatic cancer group, and metastatic cancer group, based on their cancer diagnosis and its extent, Kaplan–Meier curves were constructed among these patient groups. Furthermore, patients were divided into a survival group and a non‐survival group based on their 28‐day survival status after ICU admission. Univariate and multivariate logistic regression analyses were performed to detect the risk factors for 28‐day mortality among these patients. Additionally, this investigation sought to establish a dose–response relationship by exploring the graded association between APS‐III scores and the 28‐day mortalities among patients diagnosed with cancer.

**Results:**

A total of 42,037 medical records were screened, from which 11,461 elderly patients aged over 80 years were included, comprising 1020 (8.90%) with non‐metastatic cancer, 537 (4.68%) with metastatic cancer, and 9904 (86.41%) without cancer. Significant differences in 28‐day mortality were observed between both the non‐metastatic and metastatic cancer groups compared to the non‐cancer group (20.98% and 22.35% vs. 15.75%, *p* < 0.001). However, no statistically significant difference was detected in the 28‐day mortality rate when comparing the non‐metastatic cancer group directly with the metastatic cancer group (20.98% vs. 22.35%, *p* = 0.576). Univariate analysis revealed significant differences (*p* < 0.001) in age, gender, BMI, aCCI excluding cancer point, ventilation, presence of cancer, and status of metastatic cancer between the survival and non‐survival groups. In the multivariate logistic regression, the odds ratio (OR) for ventilation was found to be 2.154 (95% CI: 1.799–2.578), cancer conferred an OR of 1.499 (95% CI: 1.137–1.975), metastatic cancer showed an OR of 1.171 (95% CI: 0.745–1.841), APS‐III showed an OR of 1.038 (95% CI: 1.034–1.042). A dose–response relationship was observed, demonstrating that when the APS‐III score exceeded 80 points, the 28‐day mortality rate surpassed 50% among the very elderly cancer patients in ICU.

**Conclusions:**

More than one‐tenth of critically ill very elderly patients admitted to the ICU are diagnosed with cancer. Among ICU patients, those with cancer face a short‐term mortality risk approximately 1.5 times higher than those without a cancer diagnosis. Interestingly, while our findings do not indicate an escalated mortality risk due to metastasis within the cancer patient cohort, the presence of cancer itself remains a significant factor influencing ICU mortality rates in this very elderly population.

## Graphical abstract

Data from the MIMIC‐IV database included 11,461 patients aged 80 years and older. The 28‐day mortality rate was significantly higher among patients with cancer compared to those without cancer. Notably, the presence of metastasis did not confer an additional increase in mortality for cancer patients significantly. When the APS‐III score exceeded 80 points, the 28‐day mortality rate surpassed 50% among the very elderly cancer patients in ICU.

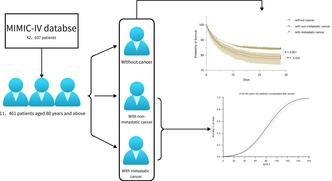

## 1 INTRODUCTION

The proportion of very elderly patients in intensive care units (ICUs) has increased along with population aging, and it is expected that by the middle of this century,^1^, ^2^ approximately 18% of ICU patients will be over 75 years old and that admission will be complicated by cancer in nearly one‐fifth of these patients.^3^ Whether very elderly patients with cancer should receive advanced life support or palliative care before ICU admission is a serious challenge that clinicians and patients must confront together.^4^ Therefore, it is necessary to identify the risk factors for death in very elderly patients with cancer and determine the extent to which each increases short‐term mortality rates.

Our retrospective study aimed to compare short‐term mortality rates among very elderly patients with metastatic or non‐metastatic cancer and those without a cancer diagnosis. Leveraging data from the Medical Information Mart for Intensive Care IV (MIMIC‐IV) database, we sought to elucidate the risk factors contributing to mortality and predict mortality outcomes using logistic regression analysis. The objective was to provide valuable insights as a reference for clinicians when making crucial clinical decisions.

## 2 MATERIALS AND METHODS

### 2.1 Patient and public involvement

Data for very elderly patients (aged ≥80 years) from 2008 to 2019 included in the MIMIC‐IV database were retrospectively screened. The establishment of this database was approved by the Massachusetts Institute of Technology (Cambridge, MA) and Beth Israel Deaconess Medical Center (Boston, MA), and consent was obtained for the original data collection. All patient‐related information from the MIMIC‐IV database is anonymous, and the requirement for informed consent was waived. The study was approved by the Beijing Hospital ethics committee (2021BJYYEC‐255‐01). The protocol of this study was registered with CCTR registration number ChiCTR2200060095.

Inclusion criteria: age ≥ 80 years old; complicated with or without cancer.

Exclusion criteria: data missed; malignant hematological disease; AIDS.

### 2.2 Methods

The patients aged 80 years and above were assigned to three groups: non‐cancer group, non‐metastatic cancer group, and metastatic cancer group, based on their cancer diagnosis and its extent. Subsequently, these patients were further divided into survival and non‐survival groups based on their 28‐day survival status after ICU admission.

Baseline data were collected within the initial 24 h of ICU admission and included age, gender, age‐adjusted Charlson comorbidity index (aCCI), aCCI excluding cancer point, body mass index (BMI), utilization of ventilation, vasopressors use, Acute Physical Score‐III (APS‐III). The outcomes were compared among the non‐cancer group, non‐metastatic cancer group, and metastatic cancer group, including 7‐day mortality, 28‐day mortality, length of ICU stay, and length of hospital stay. Additionally, 28‐day cumulative survival curves were generated for patients with metastatic cancer, non‐metastatic cancer, and those without cancer.

Univariate analysis was conducted using baseline variables, including age, gender, BMI, aCCI excluding cancer point, ventilation, vasopressor, APS‐III, presence of cancer, and status of metastatic cancer. Significant variables from the univariate analysis were further included in a multivariate logistic regression model.

Furthermore, we established the dose–response relationship between APS‐III and 28‐day mortality in cancer patients by plotting a fitted curve.

### 2.3 Statistical analysis

**FIGURE 1:**
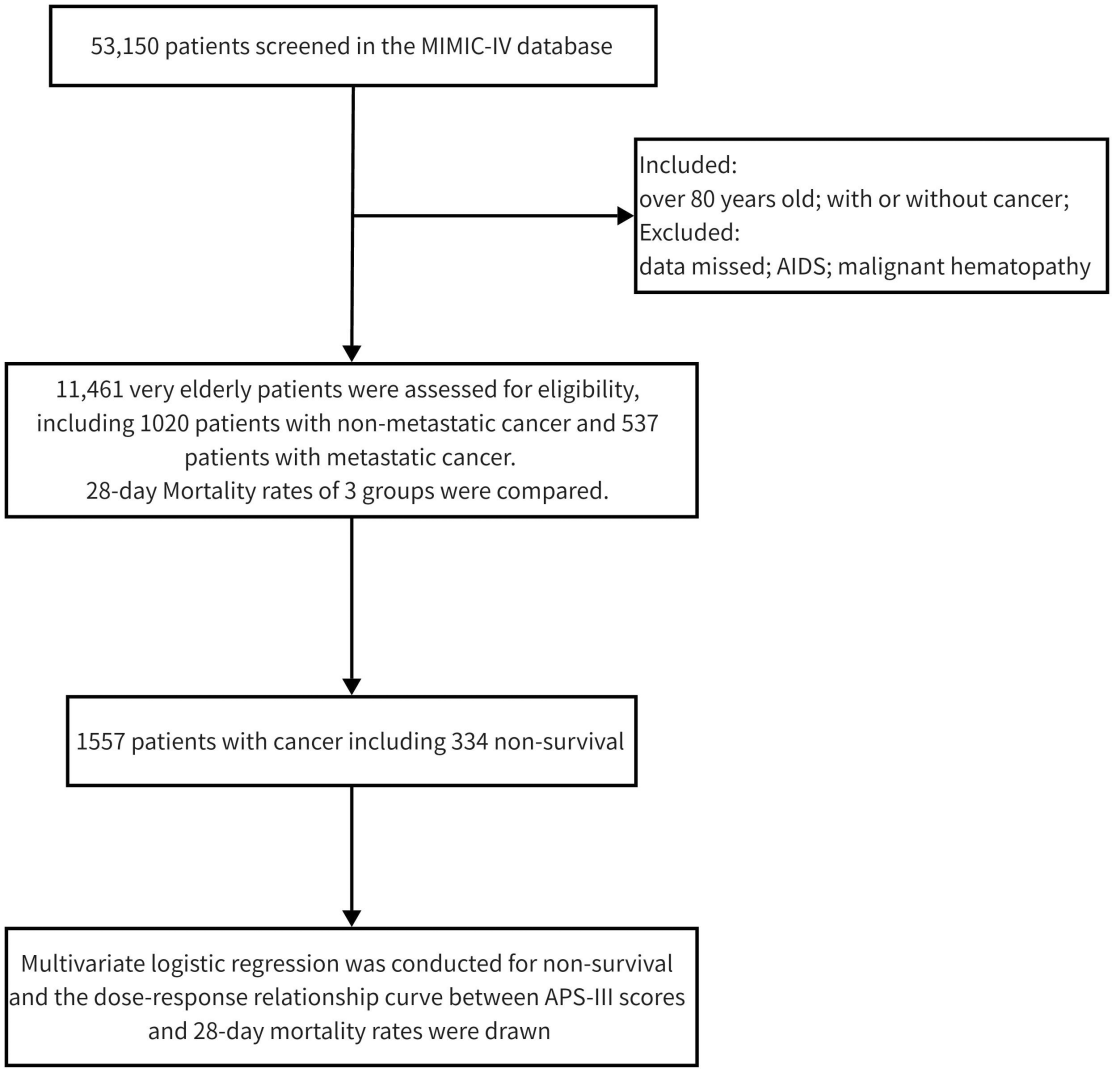
Research flow chart.

Normally distributed variables are expressed as the mean (standard deviation), whereas non‐normally distributed variables are presented as the median (interquartile range, Q1, Q3). The independent samples *t*‐test and Mann–Whitney *U*‐test were used for normally and non‐normally distributed data, respectively. Categorical variables are shown as percentages and were analyzed using the chi‐square test. ROC curves were drawn, and the cutoff value was determined according to the Youden index. Risk factors for death were analyzed using multivariate logistic regression. Statistical analyses were performed using SPSS 23.0 (IBM Corp., Armonk, NY, USA), and figures were drawn using GraphPad Prism 9.3 (GraphPad Software, San Diego, California, USA). Statistical significance was defined as *p* < 0.05 (Figure 1).

## 3 RESULTS

### 3.1 Baseline data and outcomes

**FIGURE 2:**
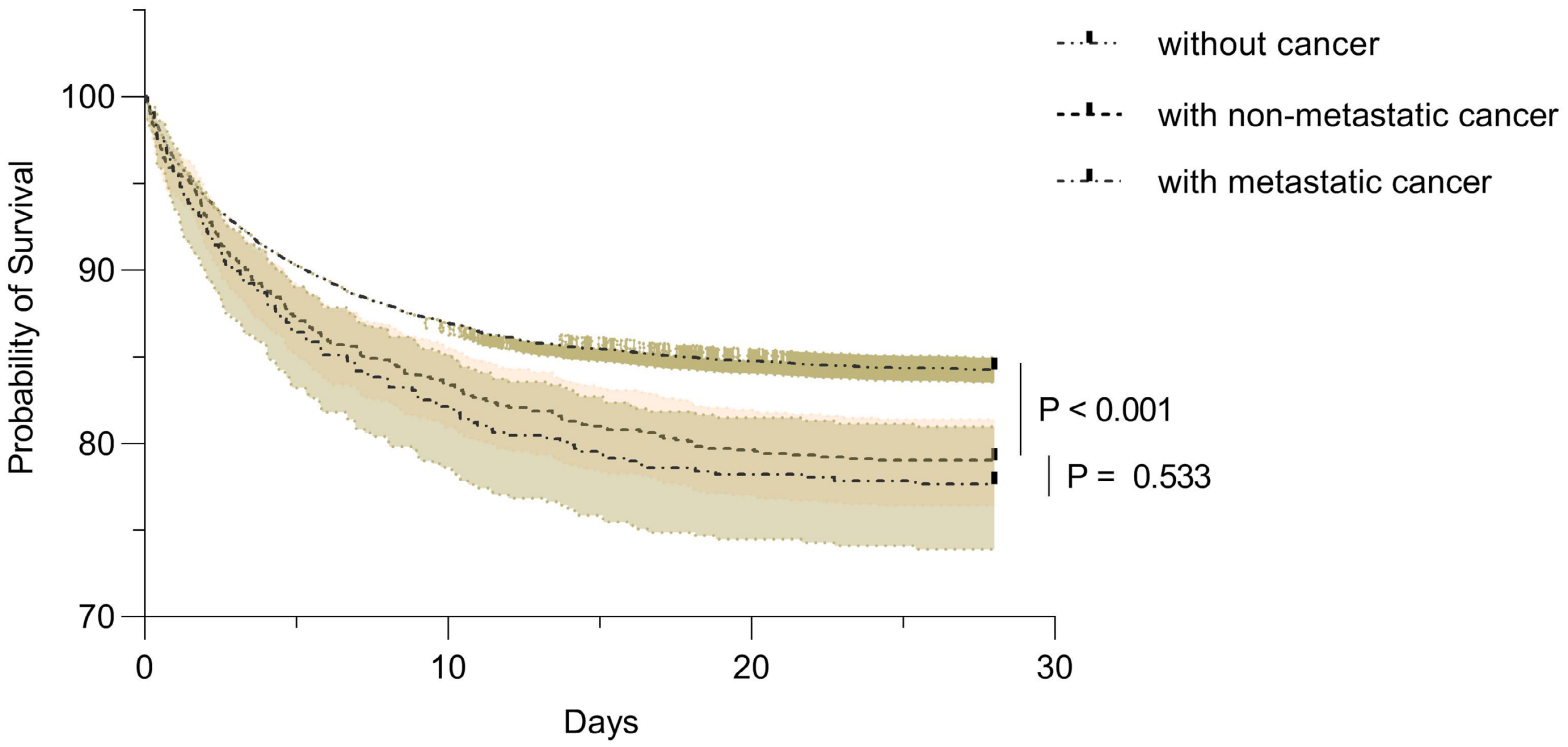
Cumulative survival curves at 28 days to ICU admission. Cumulative survival curves showed significant differences in 28‐day mortality between both the non‐metastatic and metastatic cancer groups compared to the non‐cancer group (20.98% and 22.35% vs. 15.75%, *p* < 0.001). However, no statistically significant difference was detected in the 28‐day mortality rate when comparing the non‐metastatic cancer group directly with the metastatic cancer group (20.98% vs. 22.35%, *p* = 0.533).

**FIGURE 3:**
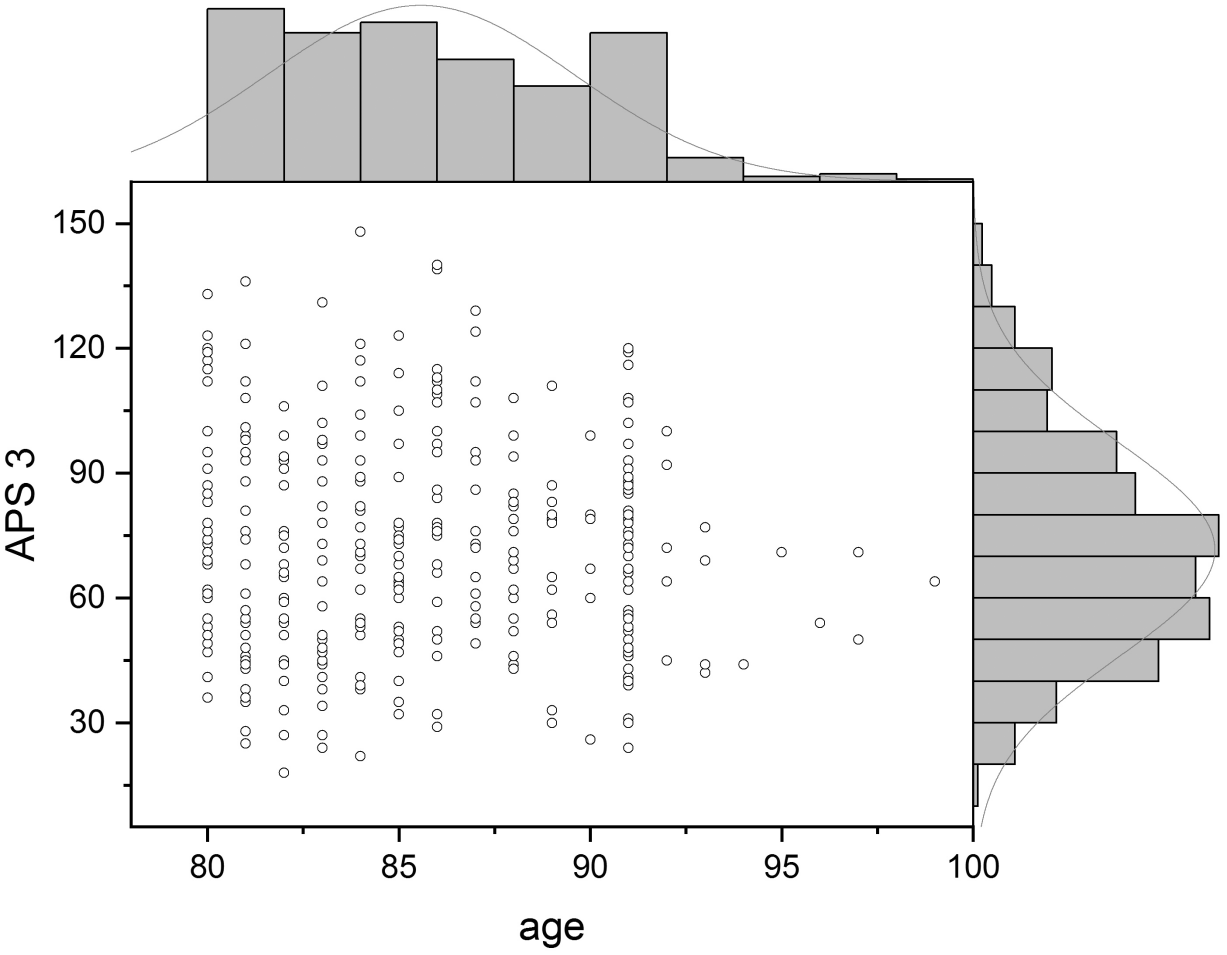
Distribution of non‐survival with cancer at different ages and APS‐III.

A total of 42,037 medical records were screened, from which 11,461 elderly patients aged over 80 years were included, comprising 1020 (8.90%) with non‐metastatic cancer, 537 (4.68%) with metastatic cancer, and 9904 (86.41%) without cancer. Significant differences in 28‐day mortality were observed between both the non‐metastatic and metastatic cancer groups compared to the non‐cancer group (20.98% and 22.35% vs. 15.75%, *p* < 0.001). However, no statistically significant difference was detected in the 28‐day mortality rate when comparing the non‐metastatic cancer group directly with the metastatic cancer group (20.98% vs. 22.35%, *p* = 0.576) (Table 1 and Figures 2 and 3).

**TABLE 1:** Baseline characteristics and outcomes of the patients.

	All	Metastatic cancer group, *n* = 537	Non‐metastatic cancer group, *n* = 1020	Non cancer group, *n* = 9904	*p* ^1^	*p* ^2^	*p* ^3^
Age, mean (SD)	85.86 (4.08)	84.85 (3.77)	85.60 (4.05)	85.94 (4.09)	<0.001	0.014	0.001
Male, *n* (%)	5315 (46.37%)	294 (54.75%)	595 (58.33%)	4426 (44.68%)	<0.001	<0.001	0.192
BMI, mean (SD)	26.55 (5.24)	25.76 (4.75)	26.46 (5.19)	26.60 (5.26)	0.068	0.595	0.109
aCCI, median [Q1, Q3]	7 [5, 8]	12 [11, 13]	8 [7, 10]	6 [5, 8]	<0.001	0.003	<0.001
aCCI excluding cancer point, median [Q1, Q3]	6 [5, 8]	6 [5, 7]	6 [5, 8]	6 [5, 8]	<0.001	<0.001	<0.001
Ventilation within 24 h, *n* (%)	3217 (28.07%)	127 (23.65%)	240 (23.53%)	2850 (28.78%)	<0.001	<0.001	0.958
Vasopressor within 24 h, *n* (%)	146 (1.27%)	8 (1.49%)	10 (0.98%)	128 (1.29%)	0.630	0.396	0.371
APS‐III within 24 h, median [Q1, Q3]	45 [35, 59]	46 [37, 62]	47 [37, 62]	44 [35, 58]	<0.001	<0.001	0.351
7‐day mortality	1365 (11.90%)	85 (15.83%)	148 (14.51%)	1132 (11.43%)	0.001	0.004	0.536
28‐day mortality	1894 (16.52%)	120 (22.35%)	214 (20.98%)	1560 (15.75%)	<0.001	<0.001	0.576
LOS in hospital (days), median [Q1, Q3]	6 [4, 10]	8 [4, 13]	7 [4, 12]	6 [4, 10]	<0.001	<0.001	0.250
LOS in ICU (days), median [Q1, Q3]	2 [1, 4]	2 [1, 3]	2 [1, 3]	2 [1, 4]	0.598	0.430	0.913

*Note*: *p* ^1^: In 3 groups; *p* ^2^: Non‐metastatic cancer group versus Non‐cancer group; *p* ^3^: Non‐metastatic group versus metastatic cancer group.

Abbreviations: LOS, length of stay; SD, standard deviation.

### 3.2 Risk factor analysis of mortality

**FIGURE 4:**
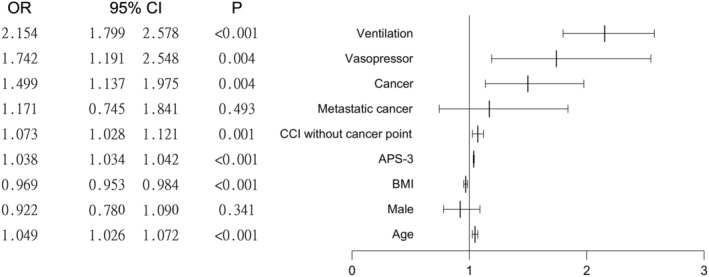
Forest plots of variables for mortality of very elderly patients. Multivariate logistic regression results identified ventilation as a significant independent risk factor with an odds ratio (OR) of 2.154 (*p* < 0.001), and vasopressor use with an OR of 1.742 (*p* = 0.004). Cancer status emerged as an independent predictor of 28‐day mortality among very elderly patients with an OR of 1.499 (*p* = 0.004). However, the presence of metastatic cancer did not reach statistical significance as an independent risk factor for 28‐day mortality, having an OR of 1.171 (*p* = 0.493).

Univariate analysis revealed significant differences in age, BMI, gender, aCCI excluding cancer point, ventilation, presence of cancer, and status of metastatic cancer between the survival and non‐survival groups (all *p* < 0.001). Multivariate logistic regression revealed the following odds ratios (ORs): ventilation, 2.154 (95% CI = 1.799–2.578, *p* < 0.001); vasopressor, 1.742 (95% CI = 1.191–2.548, *p* = 0.004); cancer, 1.499 (95% CI = 1.137–1.975, *p* = 0.004); metastatic cancer, 1.171 (95% CI = 0.745–1.841, *p* = 0.493); and APS‐III, 1.038 (95% CI = 1.034–1.042) (Table 2 and Figure 4).

**TABLE 2:** Variables of survival and non‐survival at baseline.

	Non survival, *n* = 1894	Survival, *n* = 9567	*p*
Age, mean (SD)	86.38 (4.11)	85.75 (4.07)	<0.001
Male, *n* (%)	915 (48.31%)	4400 (46.47%)	<0.001
BMI, mean (SD)	25.93 (5.41)	26.68 (5.19)	<0.001
aCCI excluding cancer point, median [Q1, Q3]	7 [5, 8]	6 [5, 8]	<0.001
With cancer, *n* (%)	334 (17.63%)	1223 (12.78%)	<0.001
With metastatic cancer, *n* (%)	240 (12.67%)	297 (3.10%)	<0.001
Ventilation, *n* (%)	930 (49.10%)	2287 (23.90%)	<0.001
Vasopressor, *n* (%)	44 (2.31%)	71 (0.74%)	<0.001
APS‐III, median [Q1, Q3]	67 [49, 88]	42 [34, 53]	<0.001

Abbreviation: SD, standard deviation.

**FIGURE 5:**
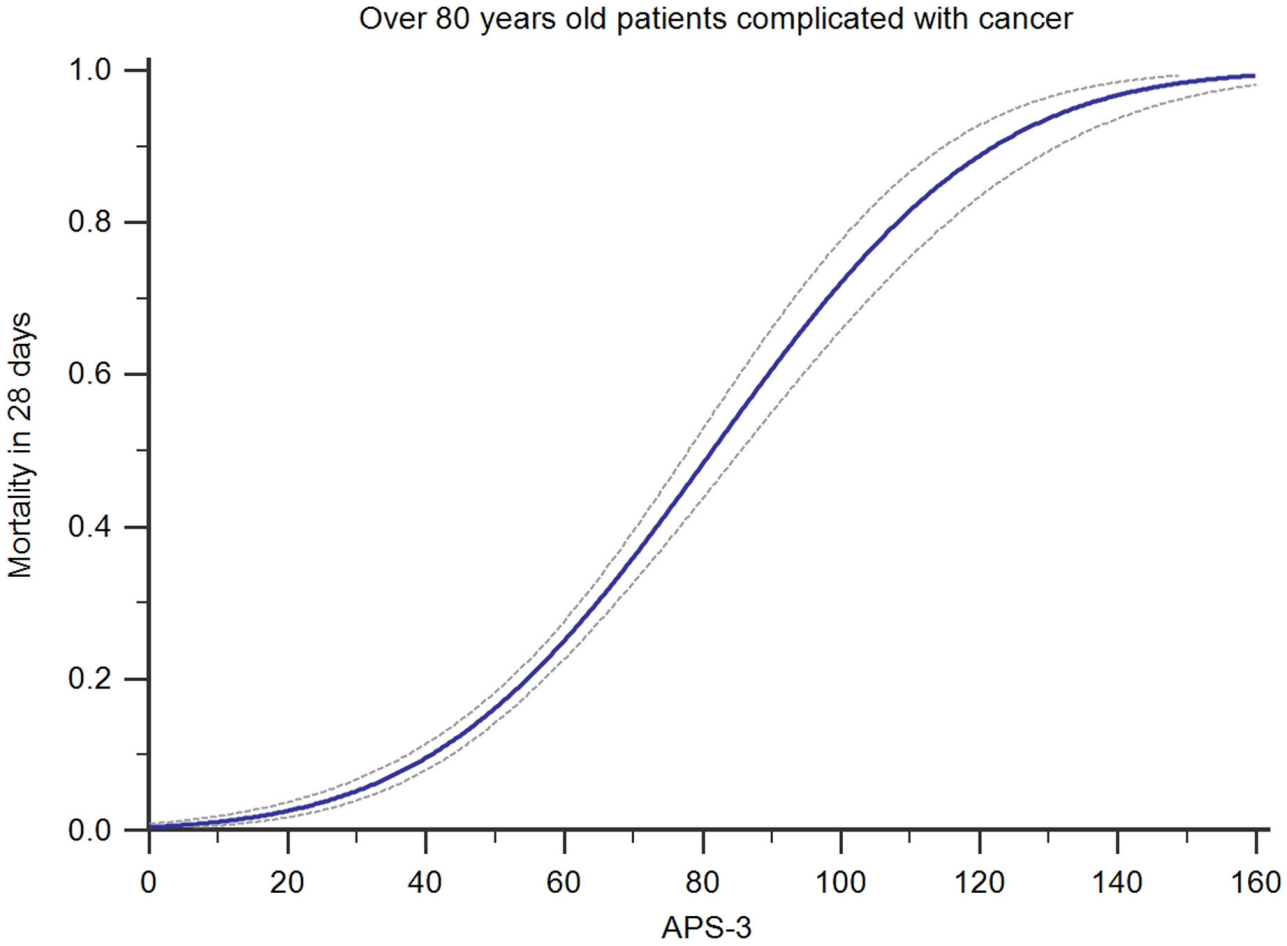
Mortality prediction for patients with cancer at different APS‐III. The dose–response curve depicted a significant correlation, indicating that the 28‐day mortality rate reached 50% when the Acute Physiology Score III (APS‐III) escalated to 80 points.

A dose–response relationship was observed, demonstrating that when the APS‐III score exceeded 80 points, the 28‐day mortality rate surpassed 50% among the very elderly cancer patients in ICU (Figure 5).

## 4 DISCUSSION

With the aging of the population structure, it has been reported that the proportion of elderly patients living in the ICU can reach more than 10%^1^ and is still increasing year by year.^5^, ^6^, ^7^, ^8^ Very elderly patients are often complicated with cancer, and these patients are transferred to the ICU due to various causes.^9^ This study found that the overall mortality of very elderly patients in the ICU could reach 16%. The short‐term mortality of patients with cancer in the ICU was significantly higher than that of patients without cancer. However, metastasis did not further increase the mortality rate among cancer patients. According to the baseline data analysis, the three groups of patients in our study were not completely consistent. Multivariate logistic regression analysis showed that cancer was an independent risk factor for short‐term mortality in very elderly individuals, with an odds ratio of 1.5.

Based on the database, we found that the short‐term mortality was significantly higher in very elderly patients complicated with cancer compared with those without cancer. However, the role of cancer in contributing to higher short‐term mortality among critically ill patients remains a subject of debate at present. Generally, the short‐term mortalities of ICU patients are mainly related to acute physiological status.^10^, ^11^ A cohort study demonstrated that the ICU mortality for patients with both cancer and sepsis aligns with their severity of organ dysfunction and is comparable to those observed in general severe sepsis and septic shock populations.^12^, ^13^ This implies that cancer do not have a dominant influence on the short‐term prognosis in ICU patients. But another large‐scale study screened over one million sepsis patients in 2019, revealing that cancer‐related sepsis carries a higher in‐hospital mortality rate than non‐cancer‐related sepsis.^14^ This finding suggests that cancer might elevate the short‐term mortality risk for critically ill patients in the ICU. As for metastasis, because of the absence of long‐term mortality in our study, we only analyzed the 28‐day mortality of patients, and there was no significant difference in the 28‐day mortality between patients with metastatic cancer and those with non‐metastatic cancer. Usually, the stage of the cancer determines the medium‐ and long‐term prognosis of patients,^15^ and the 1‐year mortality is higher in patients with metastatic tumors than in non‐metastatic patients.^16^ While some literature reports metastatic disease as a risk factor for short‐term mortality among critically ill ICU patients,^17^, ^18^, ^19^ these divergent findings could be attributed to differences in patient populations, cancer treatments, comorbidities, and other factors.

Several scoring systems, such as the Acute Physiology and Chronic Health Evaluation, SOFA, and SAPS, can predict the prognosis of critical patients.^20^, ^21^, ^22^ As these scoring tools are based on a different database, whether they have good predictive effectiveness for very elderly patients with cancer still needs to be verified. SAPS‐II is widely used for determining the severity of disease in the ICU. However, research has indicated that the AUC of the SAPS‐II score for predicting prognosis decreases with increasing age.^23^ The NEWS score is an emergency scoring system based on physical signs,^24^ while very elderly critical patients often show slight physical signs.^25^ We conducted an analysis to investigate the relationship between increasing levels of APS‐III and the 28‐day mortality rate in patients with a diagnosis of cancer. This led to the construction of a dose–response curve, which revealed that upon exceeding an APS‐III threshold of 50, the rate of mortality rises at an accelerated pace. Notably, once APS‐III attains a value of 80, the likelihood of mortality surpasses 50% among these patients. However, it is worth emphasizing that a subset of such patients may still exhibit favorable short‐term prognoses. Therefore, very elderly patients with cancer cannot be contraindications for ICU admission. When deciding whether to receive palliative treatment, these patients would refer to the information given by the clinician, including whether the disease is in the terminal stage, the prognosis estimation, and their willingness to treat.^26^

Logistic regression analysis revealed that ventilation and vasopressor treatment were important prognostic variables. Higher BMI was also identified as a protective factor for very old individuals with cancer; this may be because emaciated older patients with cancer are more likely to have cachexia and hypoproteinemia, which increase short‐term mortality.^27^, ^28^ Although the univariate analysis showed that male patients had lower mortality, this may be owing to the different distribution of several cancers, such as lung cancer, between older men and women.^29^ The significant differences disappeared in the multivariate regression analysis, likely due to the removal of multiple linear effects.

Very few patients with AIDS and hematological malignancy were excluded from the study protocol, as patients with immunosuppression exhibit differences from those with malignant solid tumors in terms of clinical characteristics and prognoses.^30^, ^31^

### 4.1 Limitations

This study has several notable limitations. First, the long‐term mortality data for these patients were not captured; this is also a critical factor in the decision‐making process of whether to pursue life‐sustaining interventions upon ICU admission for both the patients and their relatives. Second, the BMI data of some patients were missing, which may result in bias in multivariate regression, but the overall conclusion would not be changed.

## 5 CONCLUSIONS

More than one‐tenth of critically ill very elderly patients admitted to the ICU are diagnosed with cancer. Among ICU patients, those with cancer face a short‐term mortality risk approximately 1.5 times higher than those without a cancer diagnosis. Interestingly, while our findings do not indicate an escalated mortality risk due to metastasis within the cancer patient cohort, the presence of cancer itself remains a significant factor influencing ICU mortality rates in this very elderly population.

## AUTHOR CONTRIBUTIONS

Taotao Liu conceived the idea and interpreted the results, and Taotao Liu and Runyu Ding performed the analysis and drafted the manuscript. All authors read and approved the final manuscript.

## FUNDING INFORMATION

This study was supported by National High Level Hospital Clinical Research Funding [BJ‐2023‐173].

## CONFLICT OF INTEREST STATEMENT

The authors declare that they have no conflict of interests.

## ETHICAL APPROVAL

The study was approved by the Beijing Hospital ethics committee (2021BJYYEC‐255‐01).

## CONSENT TO PARTICIPATE

The requirement for informed consent was waived.

## Data Availability

The MIMIC‐IV data are available on the project website at https://mimic‐iv.mit.edu/.
